# Primary hepatic squamous cell carcinoma: case report and systematic review of the literature

**DOI:** 10.3389/fonc.2023.1229936

**Published:** 2023-10-10

**Authors:** Lin Zhao, Yan Zhou, Jianmin Ding, Zhengyi Qin, Hongyu Zhou, Xiang Jing

**Affiliations:** ^1^ Department of Ultrasound, The Third Central Hospital of Tianjin, Tianjin, China; ^2^ Tianjin Key Laboratory of Extracorporeal Life Support for Critical Diseases, Tianjin Third Central Hospital, Tianjin, China; ^3^ Tianjin Institute of Hepatobiliary Disease, Tianjin Third Central Hospital, Tianjin, China

**Keywords:** primary hepatic squamous cell carcinoma, clinical course, clinical characteristics, imaging findings, treatments and prognosis

## Abstract

Primary hepatic squamous cell carcinoma (SCC) is extremely rare, and only a few dozen cases have been reported to date. It can barely be diagnosed before histopathological examination, which necessitates the exclusion of metastatic tumors. In this case, we present a 60-year-old female patient with no comorbidity. As laboratory tests and imaging examinations were not diagnostic, ultrasonography (US)-guided liver biopsy was performed and eventually revealed a definitive pathological diagnosis of hepatic SCC. After excluding metastasis, the diagnosis of primary hepatic SCC was established, and then chemotherapy and immunotherapy were performed. Additionally, a comprehensive literature search was conducted on primary hepatic SCC using PubMed, Google Scholar, and Web of Science, and a total of 53 articles were retrieved with a time range from 1972 to 2022. A critical analysis was then performed to evaluate previous literature focusing on the clinical characteristics, imaging features, treatments, and prognosis.

## 1 Introduction

Primary hepatic squamous cell carcinoma (SCC) is extremely rare with only a few dozen cases being reported till now (1). Unlike hepatocellular carcinoma, the most common malignancy in the liver, primary hepatic SCC lacks distinctive imaging characteristics and can only be definitively diagnosed through histopathological examination, necessitating the exclusion of metastatic tumors (2). The pathogenesis of primary hepatic SCC remains unclear, which may relate to liver cysts and hepatolithiasis. However, some patients have no obvious causes (3). Currently, there is no standardized treatment approach in place for primary hepatic SCC. Surgical resection, interventional therapy, chemotherapy, and more recently immunotherapy have been employed based on individual patient characteristics; however, the majority of patients exhibit a poor prognosis with short survival (4–6). In this study, we present a case of primary hepatic SCC in an elderly patient without comorbidities. Additionally, we conducted a comprehensive analysis of relevant literature, focusing on the clinical characteristics, imaging findings, treatments, and prognosis.

## 2 Case report

In August 2020, a 60-year-old female patient was admitted to our hospital due to upper abdominal discomfort and fullness, without abdominal pain, fever, jaundice, or weight loss. Physical examination revealed a soft abdomen without tenderness, rebound tenderness, or positive Murphy’s sign. Laboratory examinations upon admission revealed leukocytosis and anemia (white blood cell (WBC) 19.79 × 10^9^/L, neutrophil 79.7%, lymphocyte 12.4%, and hemoglobin 102 g/L). Liver function tests indicated elevated alkaline phosphatase (ALP) and gamma glutamyl transpeptidase (GGT) (ALP 170 U/L and GGT 136 U/L), and other indicators (alanine aminotransferase (ALT), aspartate aminotransferase (AST), total bilirubin (TBIL), and direct bilirubin (DBIL)) were within normal range. There was no history of hepatitis B virus or hepatitis C virus infection reported by the patient. The tumor marker of SCC exhibited significant elevation, and carbohydrate antigen 19-9 (CA19-9) showed a slight increase, while alpha-fetoprotein (AFP), CA72-4, and carcinoembryonic antigen (CEA) levels were normal. C-reactive protein (CRP) level was significantly elevated, while procalcitonin (PCT) level remained normal.

**Figure 1:**
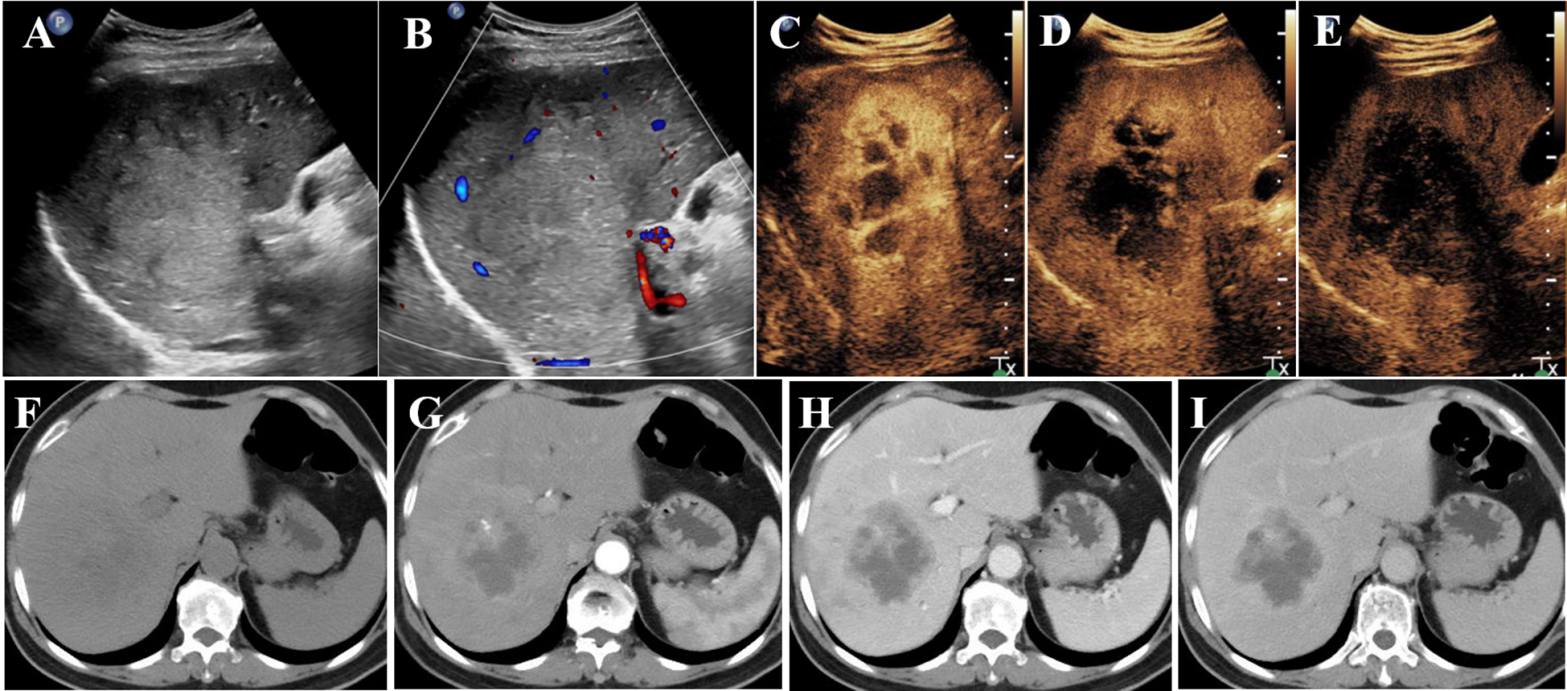
Conventional US revealed a mass **(A, B)** in the right lobe of the liver; CEUS showed irregular hyperenhancement with non-enhancement areas in the arterial phase **(C)**, then washed out early in the portal venous phase **(D)**, and finally marked washed out in the late phase **(E)**. CT revealed a heterogeneous low-density mass in the right lobe of the liver **(F)**; CECT showed mild-to- moderate heterogeneous enhancement in the arterial phase **(G)** and portal venous phase **(H)** in the mass. The enhancement degree was more obvious in the delayed stage **(I)**. US, ultrasonography; CEUS, contrast-enhanced ultrasonography; CECT, contrast-enhanced computed tomography.

**Figure 2:**
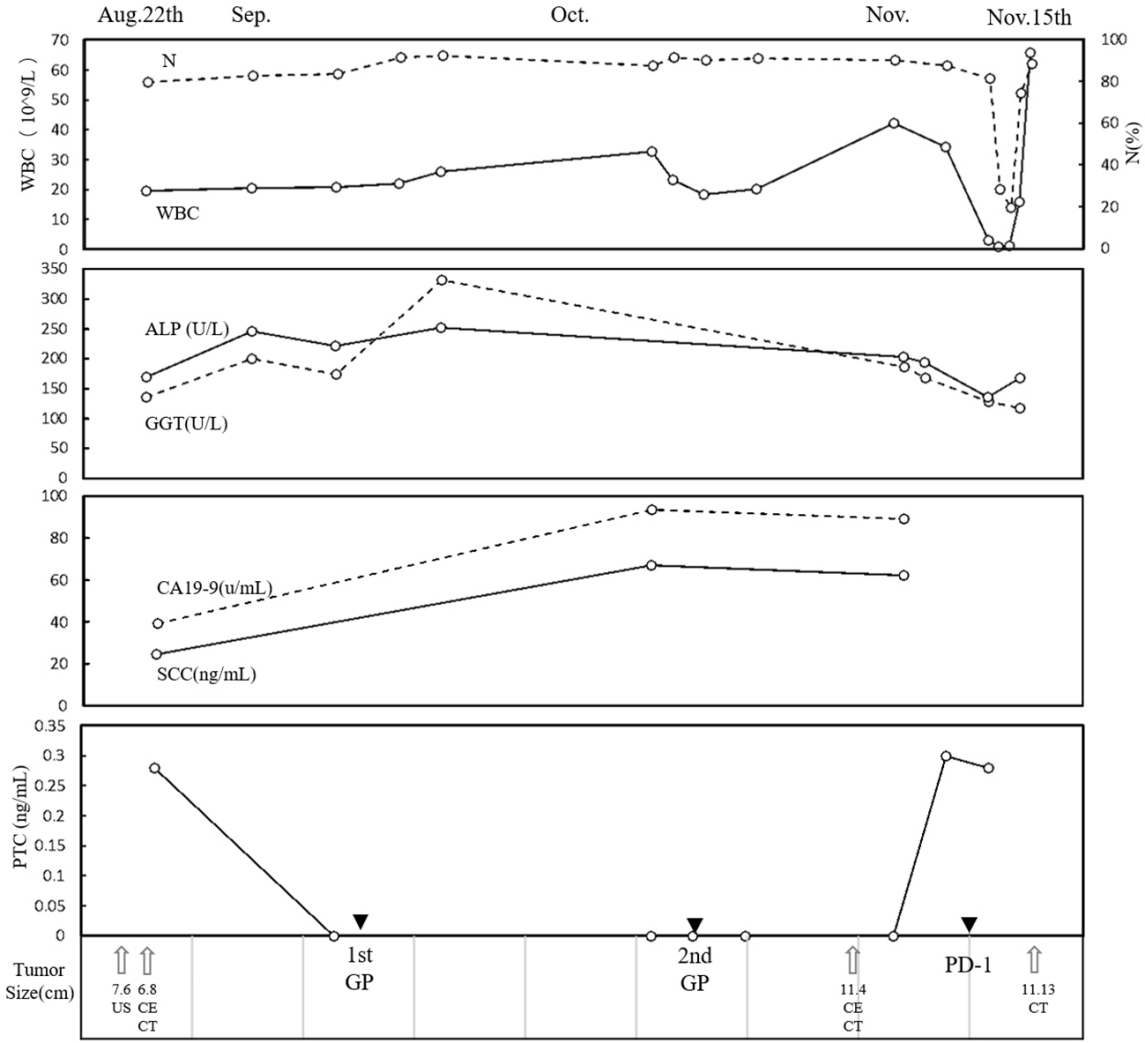
Clinical course. The time course of WBC, N, ALP, GGT, CA19-9, SCC, and PCT. Therapies and tumor size are indicated at the bottom. WBC, white blood cell; N, neutrophil; ALP, alkaline phosphatase; GGT, gamma glutamyl transpeptidase; CA19-9, carbohydrate antigen 19-9; SCC, serum squamous cell carcinoma antigen; PCT, procalcitonin.

Conventional US revealed a heterogeneous hypoechoic mass in the right posterior lobe of the liver, exhibiting an ill-defined boundary and irregular shape, which involved the right branch of the portal vein. Contrast-enhanced US (CEUS) demonstrated earlier enhancement, displaying an irregular hyperenhancement pattern with multiple non-enhancement areas within during the arterial phase, then washout early in the portal venous phase, and eventually marked washout in the late phase (**Figures 1A–E**). The abdominal computed tomography (CT) revealed a 6.3 × 6.8 cm patchy heterogeneous low-density mass with an ill-defined boundary in the right lobe of the liver, accompanied by dilation of some intrahepatic biliary ducts adjacent to the lesion in the right lobe. Contrast-enhanced CT (CECT) demonstrated mild-to- moderate heterogeneous enhancement during both arterial and portal venous phases within the mass, while the margin enhanced with a central necrotic area was devoid of enhancement. The degree of enhancement was more pronounced in the delayed phase. The mass involving segments V, VII, and VIII, the right branch of the portal vein was not clearly visualized, possibly due to tumor invasion (**Figures 1F–I**). Multiple lymph nodes were identified in the hilar region, peripancreatic region, and portacaval space, some of which demonstrated metastatic disease (**Figures 1F–I**). Positron emission tomography/CT (PET/CT) revealed no discernible lesions in the lung, gastrointestinal tract, or other organs, thus indicating a primary malignant liver tumor with lymph node metastasis. Based on the aforementioned examination, primary malignant lesions originating from intrahepatic biliary ducts were considered a possibility. In order to determine the pathology of the lesion, a US-guided liver biopsy was performed and ultimately revealed a pathological diagnosis of SCC. Immunohistochemistry analysis demonstrated CK7 (individual cells +), CK19 (partial +), Hep (−), Ki-67 proliferation index approximately 40%, CD34 (+), P63 (+), CK5/6 (+), GPC-3 (−), CK (+), and arginase-1 (−). Genetic testing revealed a PD-L1 tumor proportion score (TPS) of 1%, genomic profiling status 1 (GPS 1), tumor mutational burden (TMB) with 24.93 mutations per megabase (Mb), and microsatellite instability-high status (MSI-H). Finally, it was determined that the lesion originated from intrahepatic biliary ducts and was diagnosed as primary hepatic SCC. Due to the involvement of multiple hepatic segments, the right branch of the portal vein, and extensive lymph node metastasis, after a multidisciplinary team discussion, surgical intervention (major liver resection) was not considered the primary treatment option for this patient. She underwent treatment with gemcitabine–cisplatin (GP) chemotherapy regimen (gemcitabine 1.4 g and cisplatin 40 mg on day 1 and day 8, q21day), which is commonly used for the management of SCC in both lung and head and neck regions. Following the completion of two cycles of chemotherapy, a CECT scan was performed, revealing an increase in the size of the mass to 11.9 × 9.4 cm. According to the result of genetic testing, the treatment regimen was modified to PD-L1 combined chemotherapy (pembrolizumab 100 mg on day 1 and albumin-bound paclitaxel 400 mg on day 2, q21day). Following completion of the treatment, the patient was discharged automatically. The clinical course is shown in **Figure 2**. In July 2021, she returned to our hospital for a follow-up ultrasound examination, which revealed a reduction in tumor size to approximately 4.9 × 3.9 cm. However, unfortunately, she died of obstructive jaundice and extrahepatic metastasis 4 months later with an overall survival time of 15 months.

## 3 Methods

The literature search strategy was conducted in accordance with the Preferred Reporting Items for Systematic Reviews and Meta-Analyses (PRISMA) statement for a systematic review. The search was performed in PubMed, Google Scholar, and Web of Science with a time range from 1972 to 2022. The research strategy employed various combinations of the following terms: primary hepatic squamous cell carcinoma, ((primary squamous cell carcinoma)) AND (liver), primary hepatic SCC, (primary SCC) AND (liver). Only English-language articles with full-text availability were included in this review analysis. Two authors (L.Z. and Y.Z.) screened all manuscripts according to PRISMA guidelines, excluding duplicate records and non-relevant reports. L.Z. and Y.Z evaluated all included literature independently. Data extraction and summarization were carried out by L.Z. and Y.Z., resolving any disagreements through discussion. A total of 53 articles were included.

## 4 Discussion

Primary hepatic SCC is an exceedingly rare malignancy, with only a few dozen cases documented in the English literature worldwide. A comprehensive search was conducted on PubMed, Google Scholar, and Web of Science, yielding a total of 53 articles published between 1972 and 2022 (1, 3–54). This article aims to provide a concise overview of the clinical characteristics, imaging findings, diagnosis and treatments associated with primary hepatic SCC.

### 4.1 Clinical characteristics

In the majority of primary hepatic SCC cases, there are no identifiable risk factors for liver tumors, such as hepatitis B infection, hepatitis C infection, or cirrhosis. However, it may be associated with hepatic cysts or hepatolithiasis, but in some cases, no comorbidity was observed. The etiology of primary hepatic SCC remains elusive. It is postulated that the presence of hepatic cysts and hepatolithiasis may be associated with primary hepatic SCC, as chronic inflammation caused by infection of hepatic cysts and irritation of bile ducts by stones could potentially induce squamous metaplasia and subsequent transformation (41, 50). Ciliated hepatic foregut cysts (CHFCs) are rare cystic lesions found in the liver, which have the potential to undergo malignant transformation, leading to the development of primary SCC (55). The clinical manifestations of hepatic primary SCC lack specificity, and there are no specific indicators in laboratory examinations and imaging findings, leading to the patients being typically diagnosed at an advanced stage. Abdominal pain is the most common symptom, while less than half of the patients experience weight loss. Jaundice may be associated with liver damage or cholestasis. Laboratory tests lack specificity, and a history of hepatitis is usually absent in these patients; however, some individuals exhibit abnormalities in liver function. The levels of AFP were within normal range in all patients, while elevated levels of CEA, CA125, and CA19-9 were relatively common. However, these markers lacked specificity. Leukocytosis was observed in approximately 30% of patients, with most of them not reporting fever. This may be attributed to tumor-related leukocytosis (TRL) (48). The occurrence of TRL, a paraneoplastic syndrome, has been reported in 10%–20% of cancer patients and is associated with a poor prognosis. It has been observed that TRL does not respond well to radiotherapy or chemotherapy, leading to poor survival outcomes (56, 57). The general characteristics and treatments are summarized in **Table 1**.

**Table 1:** General characteristics and treatments.

Gender (male/female)	37/22
Weight (depression/elevation/unchanged/NM)	22/2/2/33
Abdominal pain (positive/negative/NM)	39/1/19
Jaundice (positive/negative/NM)	12/13/34
Fever (positive/negative/NM)	13/6/40
Comorbidities (liver cyst/hepatolithiasis/both/neither/NM)	22/7/2/3/19
Hepatitis (positive/negative/NM)	
HBV	2/26/31
HCV	1/19/39
Blood cell analysis	
WBC (elevation/normal/NM)	18/6/35
RBC (depression/normal/NM)	16/6/37
Liver function (elevation/normal/NM)	
ALT	13/16/30
AST	10/17/32
ALP	23/3/33
GGT	18/2/39
Bilirubin	14/14/31
Tumor marker (elevation/normal/NM)	
AFP	0/33/26
CEA	8/20/31
CA125	2/4/53
CA19-9	10/10/39
SCC	8/2/49
CYFRA 21-1	5/1/53
Treatment (surgery/intervention/non-operative treatment/NM)	31/7/17/4

NM, not mentioned; HBV, hepatitis B virus; HCV, hepatitis C virus; WBC, white blood cell; RBC, red blood cell; ALT, alanine aminotransferase; AST, aspartate aminotransferase; ALP, alkaline phosphatase; GGT, gamma glutamyl transpeptidase; AFP, alpha-fetoprotein; CEA, carcinoembryonic antigen; CA19-9, carbohydrate antigen 19-9; SCC, squamous cell carcinoma.

### 4.2 Imaging findings

The imaging findings of primary hepatic SCC lack a typical enhancement pattern, with most CT scans showing low-density heterogeneous masses, some of which may be accompanied by hepatic cysts or intrahepatic biliary duct stones. After contrast enhancement, a significant number of cases exhibit rim enhancement or delayed enhancement, resembling the patterns observed in intrahepatic cholangiocarcinoma (ICC). Differentiation from liver metastasis and liver abscess is necessary, changes in tumor markers may provide insights into the differential diagnosis. We have summarized the CECT imaging findings reported in the literature (**Table 2**). Contrast-enhanced magnetic resonance imaging (CEMRI) and CEUS were performed in a limited number of cases, yielding no typical findings. In CEMRI, the lesion may exhibit peripheral enhancement or demonstrate a high and irregular enhancement pattern (34, 46, 47). CEUS reveals heterogeneous enhancement during the arterial phase followed by washout in the late phase, indicative of malignancy (35, 44). In the present study, the patient had no history of liver disease; CECT revealed mild-to- moderate enhancement in the arterial and portal venous phases within the mass, with a more pronounced degree of enhancement observed in the delayed stage. This finding suggests an origin from the intrahepatic bile duct, consistent with previous literature reports. The CEUS demonstrated irregular hyperenhancement during the arterial phase, followed by early washout in the portal venous phase within the mass and ultimately significant washout in the late phase, resembling characteristics typically seen in ICC. Thus, the inconsistency in enhancement patterns observed between CEUS and CECT implies that, apart from ICC and vascular liver tumors, primary hepatic SCC should also be taken into consideration.

**Table 2:** Contrast-enhanced CT images of the lesions.

Authors	Years	Age/sex	CECT
Arase Y et al. (12)	1988	63/M	A large, well-circumscribed area of low density in the lower part of the right lobe of the liver. The margin of the mass was enhanced compared with the degree of enhancement within the tumor mass
Lombardo FP et al. (16)	1995	59/F	A membranelike structure was visualized at the periphery of portions of the mass, which also contained non-enhancing, frondlike, and confluent internal radiodensities. The only contrast enhancement noted was at the interface between the mass and liver parenchyma
Monteagudo M et al. (20)	1998	71/F	A right hepatic lobe mass with a large air-fluid level in which lobulated contours were enhanced
Asanuma N et al. (22)	2002	77/M	A large tumor in S8 and S7 regions of the liver, which was irregular and inhomogeneous and poorly enhanced by contrast medium
de Lajarte-Thirouard AS et al. (23)	2002	40/F	A hypodense cyst that was not enhanced
Saito T et al. (25)	2002	63/M	A lesion in the left hepatic lobe was a low-density area with a ring enhancement
Kaji R (26)	2003	67/F	The space-occupying lesion localized in the right lobe in which margin was shown by a contrast -enhanced effect in the arterial phase, and then the contrast enhancement disappeared in delayed venous phase
Yuki N et al. (30)	2006	63/M	A low-density area showing rim enhancement after contrast injection
Lee HL et al. (31)	2006	40/M	An irregular mass with inhomogeneous density and mild delayed enhancement in the central zone of the tumor near the gallbladder at S5 of right lobe of the liver
Naik S et al. (6)	2009	56/M	A hypodense mass in the left hepatic lobe near the porta hepatis with some peripheral enhancement on delayed imaging, which did not meet criteria for a benign hemangioma
Spaggiari M et al. (34)	2011	72/M	A non-homogeneous voluminous liver mass with mild contrast enhancement, located between S5 and S6 and associated with ectasic and spindle aspects of the hepatic biliary ducts
Iimuro Y et al. (35)	2011	73/F	An irregular low-density area with marginal enhancement adjacent to a non-parasitic liver cyst
Zhu KL et al. (36)	2012	46/F	A regular mass measuring with inhomogeneous density, mild delayed enhancement in the peripheral zone, necrosis in the central zone of the tumor, and dilated secondary biliary ducts with intraductal lithiasis
Wilson JM et al. (39)	2013	34/M	A hypoenhancing lesion in the right anterior sector plus left medial section of the liver
Morito K et al. (40)	2013	55/F	A mass lesion showing ring-shaped enhancement at segment 8 of the liver
Lubana SS et al. (46)	2015	64/M	An enhancing mass at the confluence of the left and right hepatic ducts
Zhang XF et al. (41)	2015	83/M	A large predominantly cystic mass in the right lobe of the liver, which was unevenly enhanced at arterial phase but slightly retrieved at venous phase
		70/F	Multiple low-density lesions in the right and left lobes, slightly uneven enhanced edge at arterial phase, further enhanced at portal venous and parenchymal phase, which was still lower than that of liver parenchyma
		50/M	Multilocular cystic lesions. The edge and interval between the lesions were slightly enhanced at arterial phase, which did not exist at portal venous
Yoo TK et al. (42)	2016	71/M	A low attenuated mass involving segment VIII and left lobe of the liver, invading left portal vein and bile duct, which showed delayed fill-in enhancement pattern
Tuminello F et al. (51)	2020	71/M	A hypodense mass in the right lobe of the liver with weakly enhanced margin
Yamada K et al. (47)	2021	52/M	A computed tomography (CT) scan with intravenous (IV) contrast revealed hypodense hepatic masses with peripheral delayed enhancement
Wang Y et al. (48)	2020	64/M	A round and ill-defined low-density region with patchy enhancement in the left lobe of liver
Xiao J et al. (1)	2021	79/M	Multiple low-density lesions with unclear boundaries and mild-to- moderate enhancement in the liver accompanied by liver cysts and gallbladder stones
		47/M	A single, big, and solid lesion with a diameter of 10 cm, with mild-to- moderate enhancement in the parenchymal part of the tumor edge
Sun Y et al. (49)	2021	73/M	There was shallow lobulation in the lesion, and during the arterial phase, the edge and the center of the lesion were mildly enhanced, and during the portal and delayed phases, the center of the lesion showed continuous enhancement
Kang LM et al. (3)	2022	73/M	An irregular mass with inhomogeneous density and moderate delayed enhancement in the left caudate liver lobe
Atiq M et al. (5)	2022	25/M	A large multilocular, multi-septate cystic lesion in segments IV, V, VI, and VII having communication with intrahepatic biliary ducts and small solid enhancing component with possibility of biliary cystadenoma
Fakhreddine O et al. (50)	2022	33/M	A mild interval increased in size in the large lobulated rim enhancing heterogeneous hepatic mass
Okuda Y (53)	2022	83/F	A low-density mass with poor contrast enhancement located in the lateral segment of the liver in the arterial phase. There was a slight contrast effect over the portal and equilibrium phases, while the center of the tumor was not contrasted

CECT, contrast-enhanced CT.

### 4.3 Treatments and prognosis

**Figure 3:**
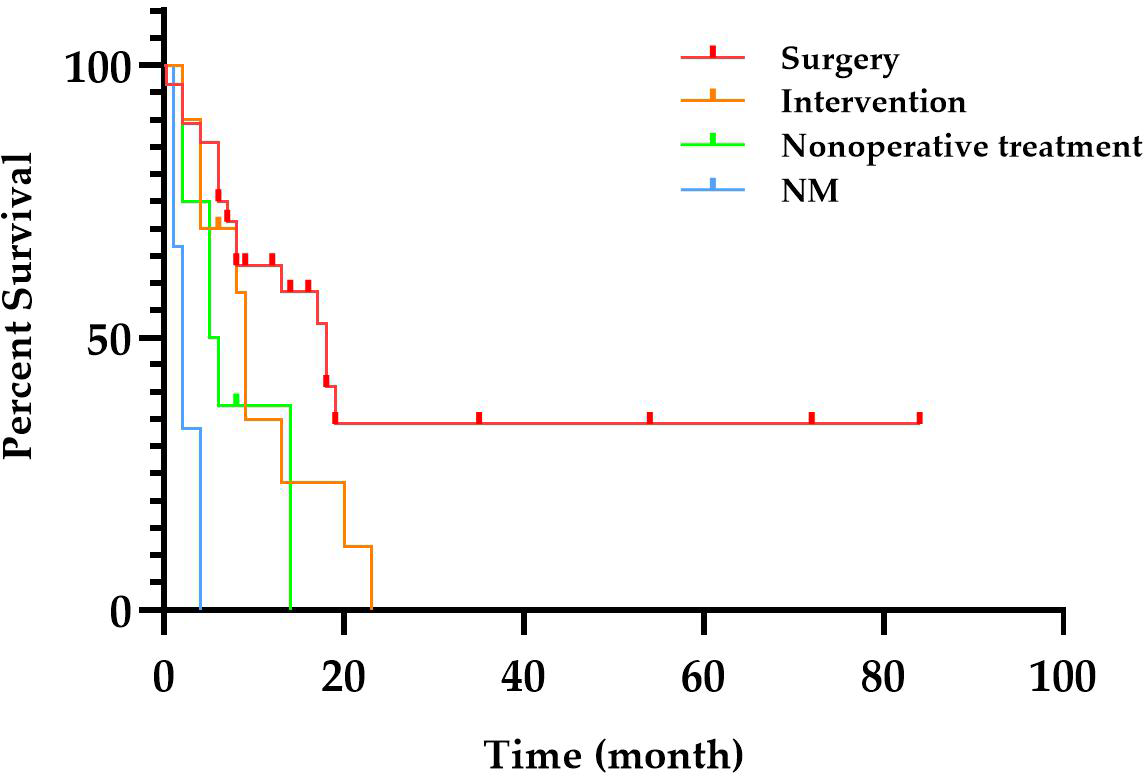
Overall survival time of primary hepatic SCC with different treatments. NM, not mentioned; SCC, squamous cell carcinoma.

The diagnosis of primary hepatic SCC necessitates a comprehensive systemic examination to exclude the presence of metastatic lesions, and the final diagnosis still requires pathological analysis and immunohistochemistry. Given the rarity of primary hepatic SCC, there is currently no standardized treatment method. Surgical intervention, interventional therapy, chemotherapy, and immunotherapy can be considered based on the patient’s overall condition and tumor characteristics (1, 4, 41, 50). We conducted an analysis of the treatment regimen and overall survival time among the patients. The patients were categorized into four groups based on different treatment modalities. Those who underwent surgery or interventional therapy (such as transarterial chemoembolization (TACE) and ablation) were assigned to either the surgery group or the intervention group. The non-operative group encompassed radiotherapy, chemotherapy, immunotherapy, or combination therapy. It should be noted that some patients’ treatments were not mentioned (NM). As shown in **Figure 3**, patients who underwent surgery exhibited better overall survival, potentially attributed to the predominance of early-stage tumors amenable to radical treatment. Conversely, patients receiving non-operative management exhibited shorter survival times due to their inability to undergo radical surgery. Additionally, primary hepatic SCC cases may exhibit resistance toward radiotherapy and chemotherapy, further contributing to a poor prognosis. In recent years, the application of immunotherapy in cancer has provided new hope for primary hepatic SCC. In our case, the patient was given an initial treatment with a GP chemotherapy regimen administered, which is commonly employed for head and neck SCC as well as lung SCC. However, the patient demonstrated a poor response to chemotherapy, resulting in tumor progression following two cycles of treatment. The patient exhibited a significantly elevated level of WBC, which may be associated with chemotherapy resistance. As genetic testing showed PD-L1 TPS 1%, TMB 24.93 mutations/Mb, GPS 1, and MSI-H, pembrolizumab, a PD-1 inhibitor, was administered due to its approval for the treatment of various cancer types when TMB is ≥10 mutations/Mb or in cases of MSI-H. Additionally, the values of TPS and GPS further support this therapeutic approach (58). A significant reduction in the size of the lesion after 1 year demonstrated the efficacy of the treatment. The combination of surgery and PD-1 inhibitors has consistently shown improved outcomes when available (3, 4). Therefore, we hypothesize that leukocytosis in patients with primary hepatic SCC may indicate resistance to chemoradiotherapy, prompting genetic testing to determine eligibility for immunotherapy.

## 5 Conclusions

In conclusion, we present a case report of primary hepatic SCC, summarizing the clinical characteristics, imaging features, treatments, and prognosis from relevant literature. Treatment options should be tailored based on the patient’s overall condition and tumor characteristics. Additionally, genetic testing may aid in determining the suitability of immunotherapy as a radical treatment alternative when conventional approaches are not feasible. This is particularly important for patients with leukocytosis, which suggests potential resistance to radiotherapy and chemotherapy.

## Data availability statement

The raw data supporting the conclusions of this article will be made available by the authors, without undue reservation.

## Ethics statement

The studies involving humans were approved by The Third Central Hospital of Tianjin Research Ethics Committee. The studies were conducted in accordance with the local legislation and institutional requirements. The participants provided their written informed consent to participate in this study. Written informed consent was obtained from the individual(s) for the publication of any potentially identifiable images or data included in this article.

## Author contributions

Conceptualization, XJ; data curation, LZ and YZ; writing—original draft preparation, LZ; writing—review and editing, YZ, JD, ZQ, and HZ; supervision, XJ. All authors contributed to the article and approved the submitted version.
